# The use of high resolution melting analysis of *ITS-1* for rapid differentiation of parasitic nematodes *Haemonchus contortus* and *Ashworthius sidemi*

**DOI:** 10.1038/s41598-020-73037-9

**Published:** 2020-09-29

**Authors:** Lucie Skorpikova, Nikol Reslova, Jan Magdalek, Jaroslav Vadlejch, Martin Kasny

**Affiliations:** 1 Department of Botany and Zoology, Faculty of Science, Masaryk University, Brno, 611 37 Czech Republic; 2 Department of Zoology and Fisheries, Faculty of Agrobiology, Food and Natural Resources, Czech University of Life Sciences Prague, Prague, 165 00 Czech Republic

**Keywords:** Molecular biology, Zoology

## Abstract

Among gastrointestinal nematodes, haematophagous strongylids *Haemonchus contortus* and *Ashworthius sidemi* belong to the most pathogenic parasites of both domestic and wild ruminants. Correct identification of parasitic taxa is of crucial importance in many areas of parasite research, including monitoring of occurrence, epidemiological studies, or testing of effectiveness of therapy. In this study, we identified *H. contortus* and *A. sidemi* in a broad range of ruminant hosts that occur in the Czech Republic using morphological/morphometric and molecular approaches. As an advanced molecular method, we employed qPCR followed by High Resolution Melting analysis, specifically targeting the *internal transcribed spacer 1* (*ITS-1*) sequence to distinguish the two nematode species. We demonstrate that High Resolution Melting curves allow for taxonomic affiliation, making it a convenient, rapid, and reliable identification tool.

## Introduction

Infection of domestic and wild ruminants by helminth parasites, especially gastrointestinal nematodes (GINs), has a considerable social and economic impact throughout the world. These infections can lead to significant economic losses both in livestock industry and wildlife ranching due to decreased productivity or even animal death^1–4^.

Among the GINs, trichostrongylid *Haemonchus contortus* belongs to the most important parasites of a wide range of small ruminants in tropical and temperate regions around the globe^5–7^. It has a direct life cycle alternating between a parasitic and a free-living stage. Hosts become infected after accidental ingestion of third-stage infective larvae (L3) during grazing. Adult nematodes attach the abomasal mucosa where they feed on host’s blood, which poses a burden on animal’s health, significant especially in sheep husbandry^8,9^. *H. contortus* is currently emerging as a model organism of anthelmintic resistance in parasites, an issue that poses an increasing problem^10–12^.

*Ashworthius sidemi* is another haematophagous abomasal nematode, phylogenetically related to *H. contortus*. It is a typical parasite of Asiatic deer that was introduced into Europe probably via the sika deer (*Cervus nippon*)^13–16^. A highly successful invasive parasite has been dynamically spreading among various species of wild ruminants and into new regions^17–20^. In the European bison (*Bison bonasus*), a new susceptible host, the intensity of infection can reach thousands of nematodes per animal, which leads to massive histopathological changes^20–22^. Some studies highlight the danger of *A. sidemi* transmission from wildlife to livestock^23–25^. Based on available evidence, it is to be feared that *A. sidemi* has the potential of becoming one of the most widespread pathogenic gastrointestinal nematodes of autochthonous European ruminants.

As noted above, GINs of domestic and wild ruminants have a negative impact on animal health, which can translate into economic losses^1–4^. The impact of infection differs depending on ruminant species, age, environment, nutrition, management, the time of year, and obviously also parasitic species and its pathogenicity, therefore the proper identification of parasitic taxa is crucial both for veterinarians and producers^25,26^.

Correct differentiation between *H. contortus* and *A. sidemi* is complicated by morphological, biochemical, and biological similarities between the two species^20,26,27^. Taxonomic identification based on morphological/morphometric features is possible, especially in adult male specimens, but in adult females and immature nematodes, these methods are unreliable and in the larval stages and eggs they are not applicable at all. Moreover, because morphological examination is time-consuming and requires a helminthologist of considerable experience, we witness a growing use of molecular tools for species identification and demand for novel approaches^28–30^.

High Resolution Melting (HRM) analysis is a PCR-based technique available for routine diagnostic applications^31–33^. It can detect sequence alterations, such as small deletions, insertions, or even single nucleotide polymorphisms (SNPs) in dsDNA fragments amplified by qPCR, which are subsequently denatured by increasing temperature, i.e. by melting. Differences in melting profiles are visualised as the fluorescence of saturating dye that is gradually disassociated from the dsDNA amplicons. HRM is a fast, simple, and cost-effective approach for genotyping and mutation scanning, and it can easily be applied to the taxonomic identification of nematodes^34–36^.

The aim of this study was to develop a fast and usable qPCR-HRM method, which uses polymorphisms in the *internal transcribed spacer 1* (*ITS-1*) region to distinguish between *H. contortus* and *A. sidemi*. This approach allowed us to evaluate the potential of HRM analysis for intravital diagnostics without any need for additional confirmation by e.g. electrophoretic separation or sequencing.

## Results

### Establishment and optimisation of HRM reference curves

#### Selection of specimens

We analysed adult males of *H. contortus* and *A. sidemi* nematodes (ten specimens from each species; 1M–20M) collected from the gastrointestinal tract of ruminants and constructed their HRM specific reference curves. Table 1 shows the range and area of origin of the host species present in the Czech Republic that were included in the study to assess possible sequential variability in the selected *internal transcribed spacer* (*ITS-1*) region. Prior to molecular analysis, we conducted a morphological identification of the male nematodes to confirm their species affiliation (see Supplementary Tables S1 and S2).

**Table 1:** Geographical origin of hosts of individual specimens of *H. contortus* and *A. sidemi* adults.

ID	Parasite		Host	Region
	Species	Sex		
1M	*A. sidemi*	Male	European bison (*Bison bonasus*)	Liberec
2M	*A. sidemi*	Male	Roe deer (*Capreolus capreolus*)	Pilsen
3M	*A. sidemi*	Male	Fallow deer (*Dama dama*)	Liberec
4M	*A. sidemi*	Male	Red deer (*Cervus elaphus*)	Central Bohemia
5M	*A. sidemi*	Male	Red deer (*Cervus elaphus*)	Pilsen
6M	*A. sidemi*	Male	Red deer (*Cervus elaphus*)	Liberec
7M	*A. sidemi*	Male	Roe deer (*Capreolus capreolus*)	Pilsen
8M	*A. sidemi*	Male	Red deer (*Cervus elaphus*)	Liberec
9M	*A. sidemi*	Male	Roe deer (*Capreolus capreolus*)	Pilsen
10M	*A. sidemi*	Male	Fallow deer (*Dama dama*)	Liberec
11M	*H. contortus*	Male	Mouflon (*Ovis musimon*)	Liberec
12M	*H. contortus*	Male	White-tailed deer (*Odocoileus virginianus*)	Moravia–Silesia
13M	*H. contortus*	Male	Roe deer (*Capreolus capreolus*)	Central Bohemia
14M	*H. contortus*	Male	Mouflon (*Ovis musimon*)	Liberec
15M	*H. contortus*	Male	Wild goat (*Capra aegagrus*)	Liberec
16M	*H. contortus*	Male	Roe deer (*Capreolus capreolus*)	Central Bohemia
17M	*H. contortus*	Male	Domestic sheep (*Ovis aries*)	South Bohemia
18M	*H. contortus*	Male	Domestic sheep (*Ovis aries*)	South Bohemia
19M	*H. contortus*	Male	Wild goat (*Capra aegagrus*)	Liberec
20M	*H. contortus*	Male	Roe deer (*Capreolus capreolus*)	Central Bohemia
1F	*A. sidemi*	Female	Red deer (*Cervus elaphus*)	Pilsen
2F	*A. sidemi*	Female	Red deer (*Cervus elaphus*)	Pilsen
3F	*A. sidemi*	Female	Red deer (*Cervus elaphus*)	Liberec
4F	*A. sidemi*	Female	Red deer (*Cervus elaphus*)	Liberec
5F	*A. sidemi*	Female	European bison (*Bison bonasus*)	Liberec
6F	*A. sidemi*	Female	European bison (*Bison bonasus*)	Liberec
7F	*A. sidemi*	Female	Moose (*Alces alces*)	Pilsen
8F	*A. sidemi*	Female	Moose (*Alces alces*)	Pilsen
9F	*A. sidemi*	Female	Roe deer (*Capreolus capreolus*)	Karlovy Vary
10F	*A. sidemi*	Female	Roe deer (*Capreolus capreolus*)	Karlovy Vary
11F	*A. sidemi*	Female	Red deer (*Cervus elaphus*)	Liberec
12F	*A. sidemi*	Female	Red deer (*Cervus elaphus*)	Liberec
13F	*A. sidemi*	Female	Fallow deer (*Dama dama*)	Liberec
14F	*A. sidemi*	Female	Fallow deer (*Dama dama*)	Liberec
15F	*A. sidemi*	Female	Fallow deer (*Dama dama*)	Liberec
16F	*H. contortus*	Female	Mouflon (*Ovis musimon*)	Liberec
17F	*H. contortus*	Female	White-tailed deer (*Odocoileus virginianus*)	Moravia-Silesia
18F	*H. contortus*	Female	Roe deer (*Capreolus capreolus*)	Central Bohemia
19F	*H. contortus*	Female	Mouflon (*Ovis musimon*)	Liberec
20F	*H. contortus*	Female	Wild goat (*Capra aegagrus*)	Liberec
21F	*H. contortus*	Female	Roe deer (*Capreolus capreolus*)	Central Bohemia
22F	*H. contortus*	Female	Domestic goat (*Capra hircus*)	Central Bohemia
23F	*H. contortus*	Female	Domestic sheep (*Ovis aries*)	South Bohemia
24F	*H. contortus*	Female	Roe deer (*Capreolus capreolus*)	Central Bohemia
25F	*H. contortus*	Female	Roe deer (*Capreolus capreolus*)	West Bohemia

#### Melting temperatures

**Figure 1:**
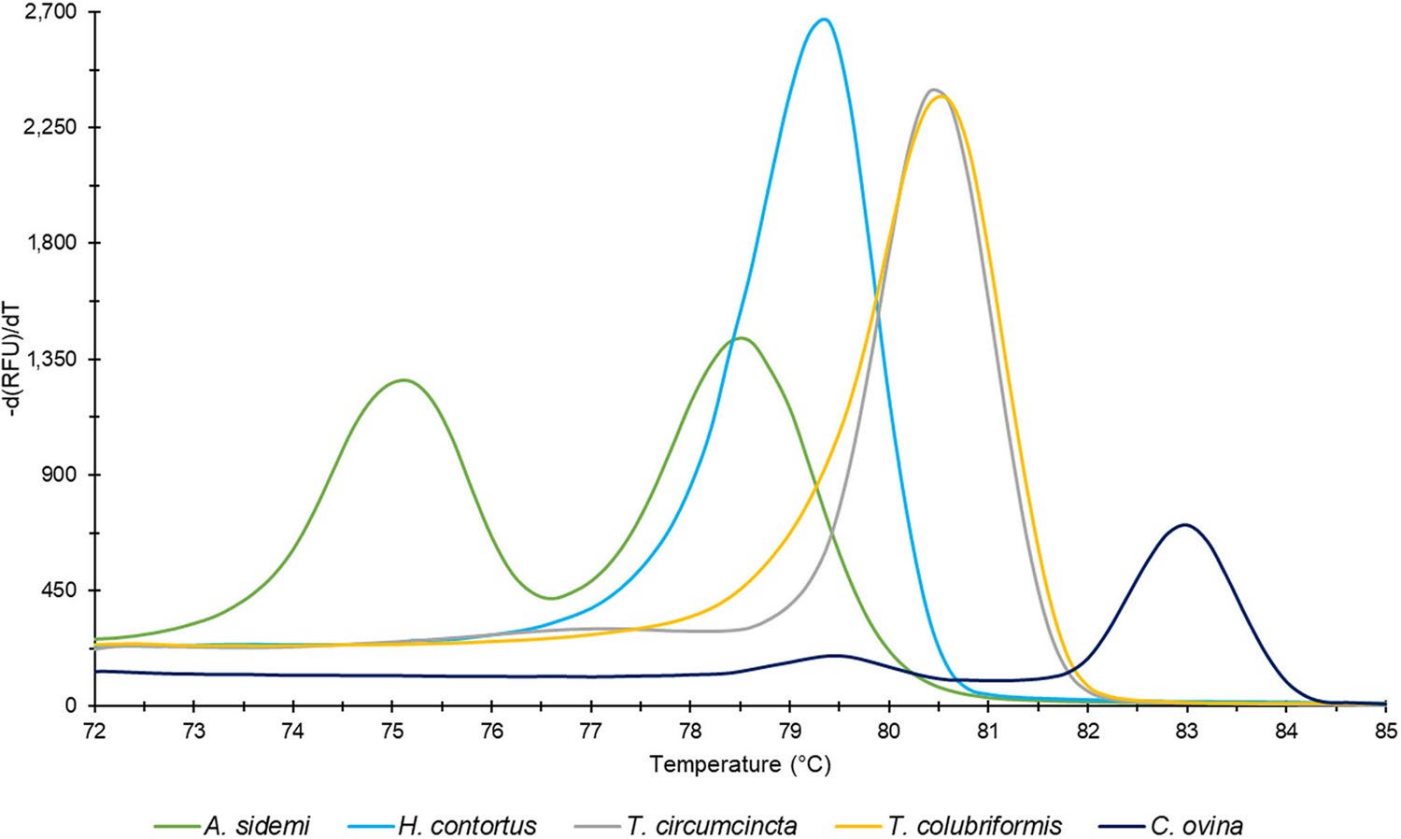
Melt peak chart. Median values of melting temperatures (Tm) of products amplified by qPCR-HRM primers corresponding to each species tested: *A. sidemi* (Tm: 75.1 °C; 78.6 °C), *H. contortus* (Tm: 79.3 °C), *T. circumcincta* (Tm: 80.5 °C), *T. colubriformis* (Tm: 80.6 °C), and *C. ovina* (Tm: 83.0 °C).

After qPCR-HRM analysis, values of melting temperatures T_m_ of amplified products with their corresponding peaks were generated by CFX Manager 3.0 software as one of the outputs. Two melting peaks at temperature mean 75.1 °C and 78.6 °C were identified as specific for the *A. sidemi* sequence, while one peak at 79.3 °C is characteristic of the *H. contortus* (Fig. 1).

#### Primer specificity validation

Primer pair specificity to the target sequence was verified using qPCR-HRM analysis of genomic DNA isolated from several related gastrointestinal nematodes recovered from ruminants. In the case of *Nematodirus battus*, *Cooperia curticei*, and *Oesophagostomum venulosum*, no products of amplification were found. In *Teladorsagia circumcincta*, *Trichostrongylus colubriformis*, and *Chabertia ovina*, products with higher T_m_ values do appear (Fig. 1) but they can easily be distinguished from the T_m_ values of *A. sidemi* and *H. contortus*.

#### qPCR-HRM analysis: normalised data and difference plot

**Figure 2:**
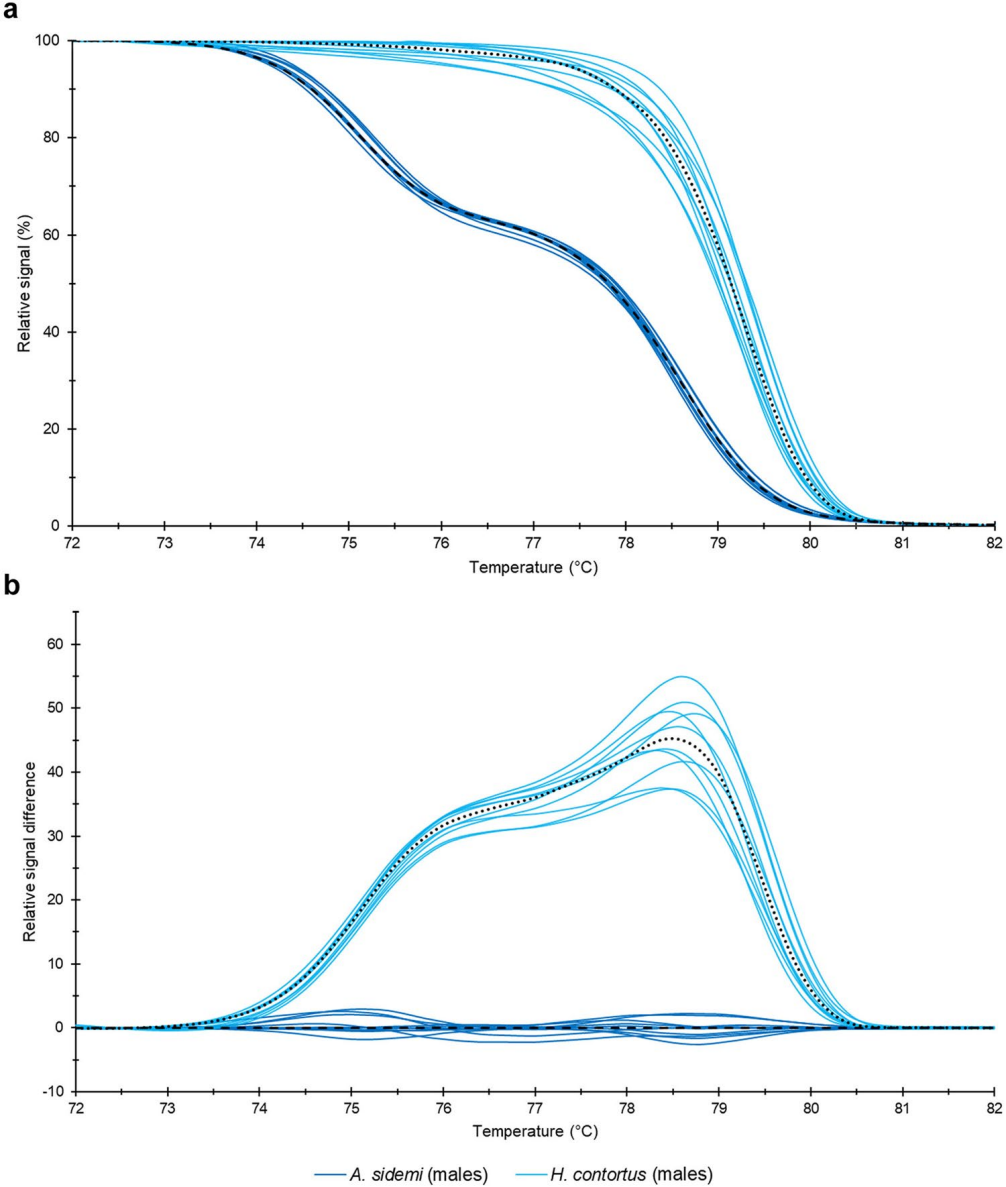
Normalised fluorescence versus temperature (**a**) and Difference plot (**b**). Data yielded by analysis of morphometrically identified *H. contortus* and *A. sidemi* adult male nematodes (samples 1M–20M). Median values are marked with a dashed line for *A. sidemi* and with a dotted line for *H. contortus*.

This analysis produced raw melting data, which were normalised and based on their shape and course of melting curves (relative fluorescence signal versus temperature) yielded two unambiguously differentiated groups corresponding to each species (Fig. 2a). To present these data as clearly as possible, we calculated from the HRM curves a difference plot (Fig. 2b).

#### Sequencing

**Figure 3:**
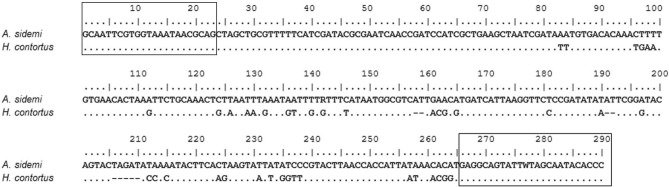
Aligned consensus sequences of the amplified *ITS-1* region. The sequences (290 bp for *A. sidemi* and 281 bp for *H. contortus*) correspond morphometrically identified *H. contortus* and *A. sidemi* adult male nematodes (samples 1M–20M). Identical nucleotides are represented by dots, gaps by hyphens. Variable sites are as indicated. Binding sites of the primers are shown in rectangles.

We amplified the *ITS-1* fragments of the male nematodes (samples 1M–20M) which were used to construct the HRM reference curves with the same primer set as above. This resulted in products of 290 bp for *A. sidemi* and 281 bp for *H. contortus* (Fig. 3). Sequencing of these products yielded identical sequences for all samples of *A. sidemi* (1M–10M) used in this study. Comparison with the GenBank sequence EF467325.1 showed that the heterozygous genotype A/G in position 142 was in all these samples conserved. *H. contortus* samples (11M–20M) all yielded a fully identical sequence, namely one corresponding to GenBank sequence AB908961.1. Results obtained by sequencing confirmed genetic conformity within each species and the relevant differences between *A. sidemi* and *H. contortus*. This strongly supports our original assumption of specificity of HRM reference curves.

### Application of qPCR-HRM analysis to other parasite forms

**Figure 4:**
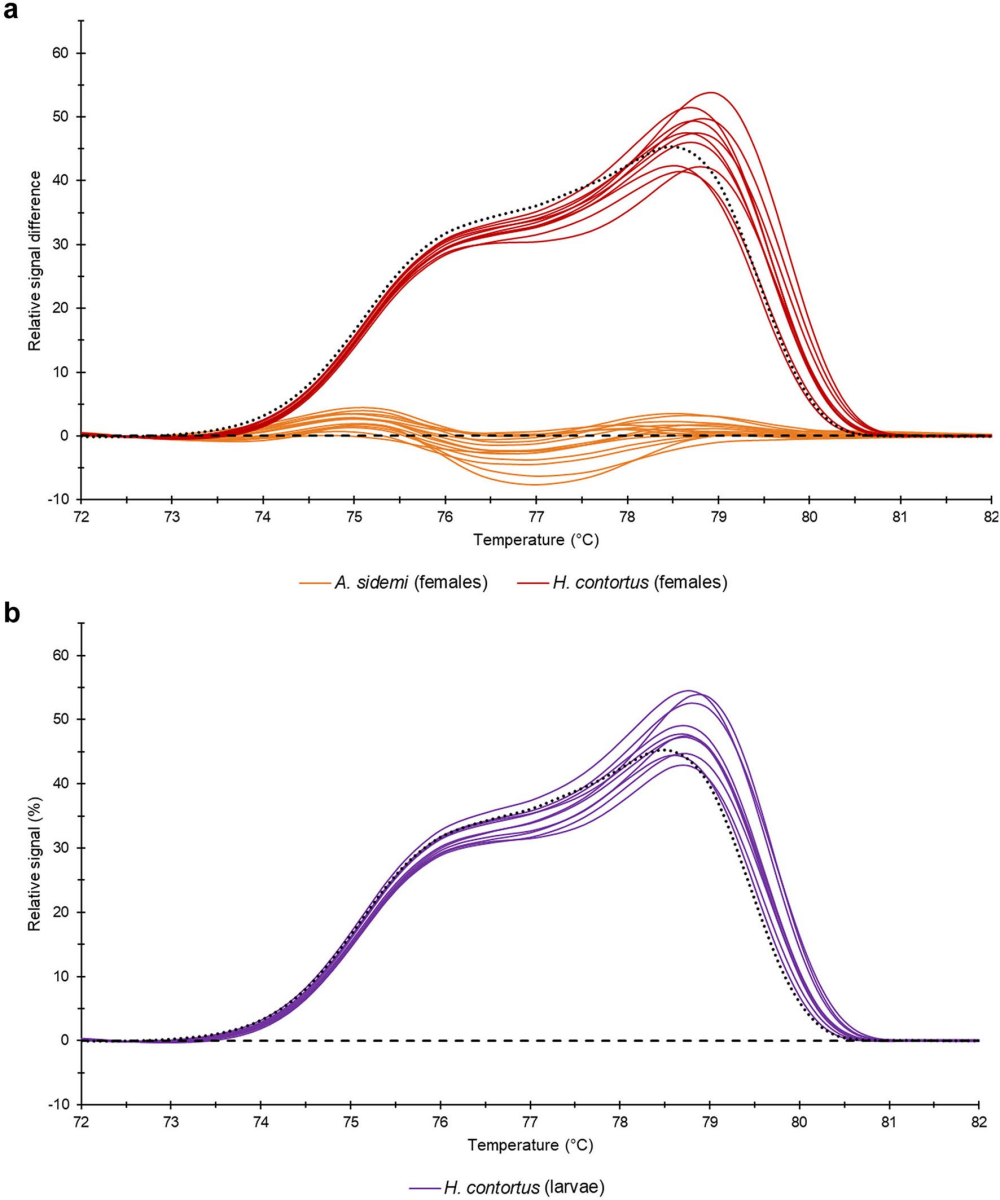
Difference plots. (**a**) data yielded by analysis of *H. contortus* and *A. sidemi* adult female nematodes (samples 1F–25F); (**b**) data yielded by analysis of *H. contortus* L3 larvae (samples 1L–10L). Median values of normalised data of male samples are marked with a dashed line for *A. sidemi* and a dotted line for *H. contortus*.

In this experiment, we identified female adult nematodes (1F–25F) using their morphological/morphometric characteristics (see Supplementary Tables S3 and S4). Then we subjected them to the same HRM analysis as the males and evaluated their data by comparing them with HRM reference curves based on male adult nematode data. All analysed samples clustered in their appropriate reference groups corresponding to the relevant species (Fig. 4a). Next, we applied HRM analysis to infective larvae of *H. contortus* (samples 1L–10L) and once again, a comparison with HRM curves based on adult male *H. contortus* showed a clear match (Fig. 4b). This suggests that the method yields correct results not only for adult male parasites but also for female adult specimens and for other developmental stages.

## Discussion

Different GINs, however, vary in their pathogenicity and if parasites are not properly taxonomically identified, nematode burdens cannot be correctly established^37,38^. Identification on at least the level of genus is crucial for mapping parasite spread, epidemiological studies, and efficacy of anthelmintic treatment.

Intravital diagnostics of GINs is still primarily based on coproscopy. Due to morphological similarities between the ‘trichostrongyle type’ eggs, the most prevalent species of nematodes in ruminants cannot be reliably differentiated, and further processing requiring faeces cultivation is needed^39^.

In the case of *H. contortus* and *A. sidemi*, the adult males can be distinguished due to differences in spicule length and presence or absence of a gubernaculum^20,26,27^. Also, adult females may be identified based on their specific morphological features, but it is often difficult to reliably differentiate between particular species.

The whole process of species differentiation using morphological characteristics is time-consuming and laborious, requires an experienced helminthologist, and is not always reliable. Therefore, molecular confirmation is appropriate if not downright necessary. For this reason, GIN diagnostics based on molecular methods is fast becoming a preferred method, because it allows for a rapid*,* safe*,* and sensitive species identification^28–30^.

Until now, multiplex PCR techniques for the detection of *A. sidemi* and *H. contortus* tended to target the nuclear ribosomal region and mitochondrial *NADH dehydrogenase subunit 4* (*ND4*)^40,41^. These approaches required a subsequent visualisation by electrophoretic separation or sequencing. The protocol designed by Lehrter et al.^40^ called for a high input of DNA (optimally 100 ng), and our experiments showed that this method is not sufficiently robust and sensitive when it comes to individual larvae (data not shown).

In the present study, we used qPCR followed by HRM analysis as an advanced molecular method to differentiate *H. contortus* and *A. sidemi* species in a one-tube trial using a single pair of universal primers. Species identification was based on *ITS-1* of the nuclear ribosomal region, a commonly used marker that sheds light on relationships between congeneric species and closely related genera of many nematodes^42–44^. For qPCR-HRM, we selected the *ITS-1* fragment, making use of the fact that this region differs between the studied species (distinct nucleotide sequences composition, length, and GC contents) and exhibits a high within-species conservation. We found that melting temperatures T_m_, and unequivocally separated HRM curves provide an easy, rapid, and unambiguous species differentiation that requires no subsequent confirmation by molecular analysis.

Furthermore, our results revealed that qPCR-HRM analysis is suitable for species identification using DNA isolated not only from adult parasites but also from other nematode developmental stages, such as infective larvae. For optimisation purposes, we used only *H. contortus* larvae (mono-infection of *A. sidemi* required for this analysis was not available during the experiment). Because individual larvae produce only a small volume of genomic DNA, we selected a suitable extraction protocol established in a previous study^36^. All in all, however, the qPCR-HRM shows promise of significant improvement of intravital GIN diagnostics that does not require post-mortem examination of host animals.

Our investigation confirmed that *H. contortus* is a generalist nematode that can infect a wide range of species of domestic and wild ruminants^45^. In the Czech Republic, the occurrence of non-native related nematode *A. sidemi* was first described in sika deer (*Cervus nippon*) in 1973^15^ and since that time, it became a widespread parasite of wild ruminants in this country^20,46^. Results of the current study also demonstrate the presence of *A. sidemi* in European bison (*Bison bonasus*), red deer (*Cervus elaphus*), roe deer (*Capreolus capreolus*), fallow deer (*Dama dama*), and moose (*Alces alces*).

Given the wide range of actual and potential host species susceptible to *A. sidemi*, it is most advisable to monitor the infection in wild and domestic ruminants precisely, because this parasite poses a considerable potential threat to naive hosts. Introduction of this parasite through European bison relocated into the Czech Republic can serve as evidence of failure of intravital diagnostics^20^. This example clearly demonstrates that accurate parasite identification is essential if we want to prevent further spread of *A. sidemi* into new areas.

## Conclusions

The results of our qPCR-HRM study based on *ITS-1* fragment of the ribosomal region allow for a rapid and reliable differentiation of parasitic nematodes *H. contortus* and *A. sidemi* on a species level without the need for electrophoretic separation of PCR products or/and sequencing. Based on specific melting curves, we identified a total of 45 specimens of adult nematodes that came from a wide range of domestic and wild ruminant species living in various parts of the Czech Republic. We also confirmed that qPCR-HRM analysis is applicable to the infective larval stages of the nematodes, which promises a significant improvement in intravital diagnostics.

## Methods

### Ethical approval

All wild ruminants were culled as part of periodic control of game populations during the hunting seasons of 2017, 2018, and 2019; domestic sheep were slaughtered for human consumption. The handling of animals was carried out in accordance with relevant guidelines and regulations valid in the Czech Republic. The moose died due to collision with a vehicle. The digestive tracts dissected in this study were provided by responsible authorities, state-owned forestry company and farmers.

### Collection of specimens

Adult nematodes were recovered from gastrointestinal tracts using post-mortem examination of ruminants; in total, we necropsied ten host species (Tab.1). The host animals came from various areas of the Czech Republic and sampling was conducted in 2017–2019. The abomasa were processed within a few hours of host culling/slaughtering using standard parasitological post-mortem examination techniques^47,48^. Organ contents were washed with tap water repeatedly and passed through mesh sieves with openings sized 200 μm and 150 μm to recover adult nematodes. Recovered worms were washed in a saline solution, preserved in 70% ethanol, and prior to morphological and molecular processing stored at 4 °C. To obtain *H. contortus* infective larvae (L3 stage), faecal samples from domestic sheep with artificial mono-infection were incubated for 7 days at 27 °C and subsequently processed using Baermann’s technique^39^. The larvae were preserved in tap water and prior to molecular processing stored at 4 °C.

### Morphological identification

The recovered nematodes were identified based on their dominant distinguishing morphological/morphometric characters as described elsewhere^20,26,27^. The individual nematode specimens were evaluated using a light microscope Olympus BX51 and measurement of their dominant morphological characteristics carried out by QuickPHOTO MICRO 3.0 software (PROMICRA).

### gDNA extraction

Molecular analysis was performed on a total of 45 adult nematode specimens morphologically identified as *H. contortus* and *A. sidemi*. In all cases, total genomic DNA was extracted separately from one half of individual adult nematodes using a slightly adjusted protocol adopted from previous studies^36,49^. The tissue was incubated in 50 μl of extraction buffer (100 mM Tris–HCl, 10 mM EDTA, 100 mM NaCl, 1% SDS, 1.5 mM dithiothreitol) containing 0.06 mg proteinase K. Lysis took place in a Thermomixer 5350 Mixer (Eppendorf) at 55 °C, with continuous mixing at 300 rpm overnight. 3 M sodium acetate (1/3 of the lysate volume), 5 μl oyster glycogen (20 mg/ml stock; SERVA Electrophoresis), and ice-cold isopropanol (2/3 of the lysate volume) were immediately added to the lysate. The sample was briefly vortexed and subsequently incubated at − 80 °C for 30 min to accelerate DNA precipitation. After centrifugation for 2 min at 14,000×*g*, the upper aqueous phase was carefully discarded. The pellet containing gDNA was washed with 200 μl of ice-cold 70% ethanol without disturbing the pellet in order to remove any salts that may be present. Another centrifugation was carried out for 2 min at 14,000×*g* and the supernatant removed. Finally, the DNA pellet was dried in a heater and dissolved in 25 μl of molecular-grade water. All samples were stored at − 20 °C until subsequent processing. gDNA was extracted from the individual larvae (n = 10) according to the same protocol, with reduction of the total extraction volume to 30 μl. In the case of larvae (6L–10L), a step of repeated thawing (at − 80 °C) and boiling (at 99 °C) was added prior to overnight lysis but no difference in DNA yields was observed. Concentration, yield, and purity of extracted gDNA were measured by NanoDrop 8000 Spectrophotometer (Thermo Fisher Scientific).

### qPCR-HRM primers design and validation

A pair of universal primers, Fw: 5′-GCAATTCGTGGTAAATAACGCAG-3′ and Rev: 5′-GGGTGTATTGCTAWAATACTGCCTC-3′ targeting *ITS-1* fragment of the ribosomal region, was used for species differentiation of *H. contortus* and *A. sidemi* nematodes that parasitised ruminants in the Czech Republic. These primers were designed according to available GenBank sequences for *H. contortus* (AB908961.1) and *A. sidemi* (EF467325.1). Basic Local Alignment Search Tool (BLAST) was used to predict primer targets in the massive GenBank database to assure both divergence among species and sequence conservation within each species. Target specificity of the primer set was verified experimentally using a qPCR-HRM analysis of related species of parasitic GINs of ruminants (*Teladorsagia circumcincta*, *Trichostrongylus colubriformis*, *Nematodirus battus*, *Cooperia curticei*, *Chabertia ovina*, and *Oesophagostomum venulosum*).

### qPCR assay and HRM analysis

The isolated genomic DNA was used as a template for qPCR, which was performed immediately prior to the HRM analysis by C1000 Touch Thermal Cycler combined with CFX96 optical module (Bio-Rad Laboratories). The mixture and conditions of the reaction were adopted from our previous study^36^. In short, the qPCR was performed in a reaction mixture comprised of 1X Kapa HRM FAST Master Mix (Kapa Biosystems) containing Eva Green saturating dye, 2.5 mM MgCl_2_, 250 nM of each primer, and 50 ng of DNA, with molecular-grade water added to a final volume of 20 μl. The qPCR cycling conditions were as follows: initial denaturation at 95 °C for 3 min, followed by 45 cycles of denaturation at 95 °C for 5 s, annealing/extension at 57 °C for 40 s, and a final cooling step at 40 °C for 30 s. Following the qPCR, amplicon dissociation was initiated by a melting step in the same machine. Temperature range was set at 70–87 °C, with a data increment of 0.2 °C per 10 s. All samples were tested in duplicate.

### Data analysis

Melting temperatures T_m_ of samples were evaluated by CFX Manager 3.0 software (Bio-Rad Laboratories). For subsequent evaluation, raw data representing the sample melting curves were extracted from this software. Data normalisation to uniform relative values from 100 to 0% for each sample was carried out by a mathematical conversion^50^ and average values calculated from duplicates. Pre- and post-melt fluorescent signals were set to 71.0–73.0 °C and 83.0–85.0 °C. To evaluate the melting process, we constructed a difference plot as an optimally transparent expression of the melting curves. First, we chose the median value of normalised data of male *A. sidemi* adult worms (samples 1M–10M) as a baseline. Then we subtracted from this baseline normalised data of all samples. Finally, we evaluated the data obtained from female adult worms (samples 1F–25F) and larvae (samples 1L–10L) according to HRM reference curves established on the basis of adult male worm data.

### DNA sequence analysis

To verify that the HRM reference curves correspond to morphometrically identified specimens of male worms (samples 1M–20M), we sequenced the gene region used to establish the HRM matrix curves. PCR products were purified using a MinElute PCR Purification Kit (Qiagen) and sequenced. All sequences were compared with the NCBI GenBank nucleotide database using the BLAST.

## Supplementary information

Supplementary Information.

## Data Availability

All data generated or analysed during this study are included in this article and its Supplementary Information file.
