# Supplementary material for: The use of high resolution melting analysis of ITS-1 for rapid differentiation of parasitic nematodes Haemonchus contortus and Ashworthius sidemi
Source: Sci Rep. 2020 Sep 29;10:15984. doi: 10.1038/s41598-020-73037-9 (PMC7525508; doi:10.1038/s41598-020-73037-9)
Supplement: Supplementary file 1 — Supplementary Information. [file 41598_2020_73037_MOESM1_ESM.pdf]

The Use of High Resolution Melting Analysis of *ITS-1* for Rapid Differentiation of Parasitic Nematodes *Haemonchus contortus* and *Ashworthius sidemi*

Lucie Skorpikova[1,*], Nikol Reslova[1], Jan Magdalek[2], Jaroslav Vadlejch[2] & Martin Kasny[1]

[1]Department of Botany and Zoology, Faculty of Science, Masaryk University, Brno, 611 37, Czech Republic

[2]Department of Zoology and Fisheries, Faculty of Agrobiology, Food and Natural Resources, Czech University of Life Sciences Prague, Prague, 165 00, Czech Republic

*skorpikovaLucie@seznam.cz

Table S1: Dominant distinguishing characters of *A. sidemi* males used in the present study.

ID	Body length (mm)	Oesophagus length (mm)	Maximal oesophagus width (mm)	Bursa copulatrix length (mm)	Gubernaculum	Spicules length (mm)*
1M	19.3	1.8	0.279	0.547	—	0.760
2M	14.2	1.6	0.322	0.472	—	0.808
3M	14.6	1.4	0.249	0.408	—	0.816
4M	15.4	1.5	0.253	0.503	—	0.766
5M	16.1	1.6	0.242	0.465	—	0.809
6M	13.4	1.5	0.252	0.600	—	0.826
7M	26.1	1.9	0.249	1.250	—	0.809
8M	25.7	1.9	0.246	1.060	—	0.808
9M	25.1	1.9	0.321	1.256	—	0.775
10M	14.6	1.4	0.296	0.408	—	0.816

* Specific identification character

Table S2 Dominant distinguishing characters of *H. contortus* males used in the present study.

ID	Body length (mm)	Oesophagus length (mm)	Oesophagus length (%)	Gubernaculum length (mm)	Spicules length (mm) *	Spicule barb length - right/left (mm)
11M	16.4	1.5	8.9	0.214	0.429	0.042/0.024
12M	13.9	1.2	8.9	0.195	0.391	0.038/0.021
13M	13.3	1.3	9.8	0.202	0.407	0.042/0.022
14M	13.8	1.3	9.2	0.240	0.420	0.045/0.024
15M	13.8	1.2	8.9	0.232	0.464	0.048/0.027
16M	13.8	1.3	9.7	0.224	0.435	0.043/0.022
17M	11.8	1.3	10.8	0.216	0.418	0.039/0.022
18M	15.5	1.4	8.8	0.228	0.476	0.042/0.024
19M	13.7	1.4	10.2	0.238	0.468	0.045/0.024
20M	12.7	1.2	9.8	0.231	0.463	0.043/0.021

* Specific identification character

Table S3: Dominant distinguishing characters of *A. sidemi* females used in the present study.

ID	Body length (mm)	Oesophagus length (mm)	Maximal oesophagus width (mm)	Shape of vulvar flap	Distance of vulva from posterior end (mm)	Distance of anus from posterior end (mm)
1F	18.4	0.170	1.6	linguiform	2.348	0.496
2F	17.7	0.144	1.6	knopped	2.093	0.513
3F	35.0	0.244	2.0	linguiform	4.480	0.573
4F	37.4	0.200	2.0	linguiform	4.437	0.562
5F	18.4	0.133	1.8	linguiform	2.100	0.476
6F	18.4	0.189	1.7	linguiform	2.170	0.498
7F	19.2	0.170	1.7	knopped	2.321	0.527
8F	17.3	0.163	1.5	knopped	2.179	0.510
9F	30.4	0.201	1.8	linguiform	4.883	0.569
10F	39.3	0.183	1.9	linguiform	4.538	0.573
11F	32.6	0.204	1.9	smooth	4.224	0.697
12F	34.6	0.235	1.8	linguiform	4.356	0.510
13F	17.5	0.198	1.7	linguiform	2.265	0.505
14F	17.4	0.175	1.5	linguiform	2.312	0.583
15F	16.2	0.182	1.5	linguiform	2.098	0.551

Table S4: Dominant distinguishing characters of *H. contortus* females used in the present study.

ID	Body length (mm)	Oesophagus length (mm)	Oesophagus length (%)	Shape of vulvar flap	Vulva length (mm)	Distance of anus from posterior end (mm)
16F	14.6	1.3	8.90	linguiform	0.893	0.396
17F	16.1	1.4	8.76	linguiform	1.223	0.443
18F	19.8	1.5	7.47	linguiform	1.172	0.478
19F	21.3	1.4	6.62	linguiform	1.044	0.424
20F	23.4	1.4	5.98	knobbed	1.118	0.439
21F	21.9	1.4	6.26	knobbed	1.080	0.416
22F	22.1	1.4	6.11	smooth	1.187	0.421
23F	16.7	1.3	7.78	knobbed	1.282	0.366
24F	18.5	1.2	6.65	knobbed	0.599	0.361
25F	22.7	1.4	5.99	linguiform	0.939	0.492
